# Concerns about the feasibility of using “precision guided sterile males” to control insects

**DOI:** 10.1038/s41467-019-11616-9

**Published:** 2019-09-02

**Authors:** Jérémy Bouyer, Marc J. B. Vreysen

**Affiliations:** 1 Insect Pest Control Laboratory, Joint FAO/IAEA Programme of Nuclear Techniques in Food and Agriculture, A-1400 Vienna, Austria; 2 Unité Mixte de Recherche ASTRE, CIRAD, INRA, Univ Montpellier, Montpellier, France

**Keywords:** Biological techniques, Biotechnology, Genetics, Molecular biology

**Arising from** N. P. Kandul et al. *Nature Communications* 10.1038/s41467-018-07964-7 (2019).

In their paper entitled “Transforming insect population control with precision guided sterile males with demonstration in flies”^1^, Kandul et al. present an innovative approach to generate sterile male insects by crossing a Cas9 line with a gRNA line. Their approach is demonstrated in the laboratory using *Drosophila melanogaster*. However, the interpretation of their results and extrapolation as a control tactic to suppress mosquito populations builds on severe inaccuracies that oversell the technology and make the paper misleading to readers.

The authors compare a competitiveness of 78% measured in “fly vials” in the laboratory for their “precision guided sterile males” to a competitiveness of 5% obtained for the RIDL (release of insects carrying a dominant lethal) approach. The latter figure was however obtained in the field^2^ and the same strain had a competitiveness of 100% against various field strains under semi-field conditions^3^ which are more realistic than “fly vials”. Similarly, a competitiveness of 72–100% was observed for irradiated male *Aedes albopictus* under semi-field conditions^4^ and of 40–80% in another study^5^. These data contradict their claim that “these results suggest that pgSIT has greater potential to eliminate local *Ae. aegypti* populations than currently available suppression technologies.”, especially in view of their lack of field data. In the sterile insect technique (SIT), most of the reduction in quality of the irradiated males is not related to irradiation *per se*, but mostly to the mass-rearing, handling and release processes of the sterile males^6^, and this will not be different for the “precision guided sterile males”. Obviously, excessive irradiation will impair competitiveness of the insects, but it is in general possible to select a trade-off dose obtaining >99% sterility of the males without significantly impacting their biological quality. As an example, irradiation doses up to 40 Gy and 90 Gy did not impair flight ability of *Ae. albopictus* and *Ae. aegypti*, respectively^7^. Moreover, 40 Gy irradiated triple *Wolbachia* infected male *Ae. albopictus* showed a competitiveness of 97% ± 26% under semi-field conditions, which was similar to untreated control males, and the release of these males successfully suppressed two isolated target populations in China^8^. However, treating male *Ae. albopictus* with a dose of 50 Gy reduced their competitiveness to 68% ± 18%.

The production of “precision guided sterile males” is based on crossing two lines and this entails the mass-rearing of two separate lines in the same factory, which is already challenging in view of the constant risk of contamination. More importantly, it requires the ability to accurately separate the males from the first line and the same for the females from the second line, and this would require the availability of a perfect sexing system. As this is currently not available, males from the first line will be contaminated with a small proportion of females of the same line, and vice versa for the second line. Mating of males and females from the same line will result in fertile offspring that will propagate the transgene in nature. If it was possible to sort the males without female contamination, would it not be more logic to use a wild strain, irradiate the males and release them since the claimed difference of competitiveness is only hypothetic?

At the time of writing, a perfect sexing system to separate the female from male mosquitoes does not exist, the best one resulting in a female contamination of 0.1%^9^. Any male or female contamination of the strains before the last crossing step to produce the “eggs for release” will result in dispersing insects of the pure Cas9 line and/or gRNA line in the environment, leading to the escape of transgenes in the wild population like with the criticized RIDL strategy.

The authors propose to “release eggs into the environment to suppress target populations” of *Ae. aegypti*. Given that this species oviposits their eggs in uncountable small breeding sites like tree holes, flowerpot saucers, crevices in rocks, and other small volume containers^10^, usually <200 mL, the distribution of the eggs into these larval habitats seems logistically impossible. If the eggs are only distributed in large, known breeding sites like water containers, it is very unlikely that the males will be able to compete with wild males given that their mean dispersal distance is <100 m (see, for example, ref. ^11^). The SIT is not simply the release of sterile males: these need to be released area-wide to ensure that they will be able to compete with the wild males over the entire target area and therefore, they need to aggregate in the very same places as the wild males^12^. An area-wide distribution would never be obtained using the release of eggs. Even the use of artificial containers containing the eggs would need to be placed every 50 m in 2 dimensions which would be cost-prohibitive in terms of man-power and other logistics. It should not be neglected that this approach would provide additional breeding sites for the wild mosquito population (potentially favoring an increase in their population size in urban areas). In addition, the initial estimated fertility of pgSIT cannot be used to predict the effectiveness of the technology as it does not take into account the ability of laboratory-reared eggs to adapt (or not) to different natural environmental conditions nor the resulting larvae to compete with wild ones.

The SIT is a method of pest control using area-wide inundative releases of sterile insects to reduce reproduction in a field population of the same species. In this respect, releasing “precision guided sterile males” can be considered as the SIT, but we would like to emphasize that the regulatory status of precision guided sterile males will be totally different than that of irradiated sterile males.

According to the Cartagena Protocol on Biosafety to the Convention on Biological Diversity, the definition of “Living modified organism” include sterile organisms. However, irradiated sterile male insects are exempted from GMO (genetically modified organism) regulations as these are products obtained by mutagenesis techniques that have conventionally been used in a number of applications, have a long safety record and do not involve the use of recombinant nucleic acid molecules^13^. Therefore, releasing “precision guided sterile males” will have to be subjected to a complex regulatory process following GMO regulations.
